# Preparation of Polybenzimidazole Hollow-Fiber Membranes for Reverse Osmosis and Nanofiltration by Changing the Spinning Air Gap

**DOI:** 10.3390/membranes8040113

**Published:** 2018-11-19

**Authors:** Xiao Wang, Palitha Jayaweera, Radwan A. Alrasheed, Saad A. Aljlil, Yousef M. Alyousef, Mohammad Alsubaei, Hamad AlRomaih, Indira Jayaweera

**Affiliations:** 1 SRI International, 333 Ravenswood Avenue, Menlo Park, CA 94025, USA; xiao.wang@sri.com (X.W.); palitha.jayaweera@sri.com (P.J.); 2 National Center for Water Treatment and Desalination Technology, King Abdulaziz City for Science and Technology, P.O Box 6086, Riyadh 11442, Saudi Arabia; ralrasheed@kacst.edu.sa (R.A.A.); saljlil@kacst.edu.sa (S.A.A.); yalyousef@kacst.edu.sa (Y.M.A.); malsubaei@kacst.edu.sa (M.A.); halrumayh@kacst.edu.sa (H.A.)

**Keywords:** polybenzimidazole, hollow fiber, air gap, reverse osmosis, nanofiltration

## Abstract

High-performance polybenzimidazole (PBI) hollow-fiber membranes (HFMs) were fabricated through a continuous dry-jet wet spinning process at SRI International. By adjusting the spinning air gap from 4″ (10.2 cm) to 0.5″ (1.3 cm), the HFM pore sizes were enlarged dramatically without any significant change of the fiber dimensional size and barrier layer thickness. When fabricated with an air gap of 2.5″ (6.4 cm) and a surface modified by NaClO solution, the PBI HFM performance was comparable to that of a commercial reverse osmosis (RO) HFM product from Toyobo in terms of salt (NaCl) rejection and water permeability. The PBI RO HFM was positively surface charged in acidic conditions (pH < 7), which enhanced salt rejection via the Donnan effect. With an air gap of 1.5″ (3.8 cm), the PBI HFM rejected MgSO_4_ and Na_2_SO_4_ above 95%, a result that compares favorably with that achieved by nanofiltration. In addition, the PBI HFM has a defect-free structure with an ultra-thin barrier layer and porous sublayer. We believe PBI HFMs are ideal for water purification and can be readily commercialized.

## 1. Introduction

Polybenzimidazole (PBI) is an attractive candidate for hollow-fiber (HF) membrane separation because of its extremely high continuous operating temperature (as high as 250 °C) [1], robust mechanical stability [2], and outstanding chemical resistance [3,4,5]. At present, spiral-wound modules based on flat-sheet membranes have dominated the market for large desalination plants, with Toyobo providing HF modules derived from cellulose triacetate (CTA) [6]. In addition to the comparable desalination performance, HF modules are superior to spiral-wound modules because they have a self-supporting structure, high packing density, and do not require a sophisticated spacer [7]. 

Although the use of PBI for reverse osmosis (RO) filtration was first described decades ago [8], there are no recent reports detailing progress in this area of use. PBI is known to be robust and has excellent desalination capabilities [9], making it an ideal candidate for RO filtration because it can withstand intensive chemical cleaning and is suitable for long-term use. As such, PBI HFMs can ensure a consistent supply of high-quality water from raw water of varying qualities.

PBI hollow fibers (HFs) may be spun by dry-jet wet spinning technology. For gas separation and desalination, HF modules are competitive with spiral-wound modules in terms of cost and efficiency [10]. A recently published review describes the use of PBI HF membranes for gas separation [11,12], nanofiltration (NF) [13], forward osmosis (FO) [2], pressure-retarded osmosis (PRO) [14] and pervaporation [15]. SRI International (SRI) has achieved continuous high-quality PBI fiber spinning at a rate of 10 km/day. Currently, we are developing a versatile spinning line to commercialize the PBI HFs and have realized a series of fiber products with different separation performance that can be made by merely changing a few key HF spinning parameters. The HF separation performance and its applications (for gas separation, RO, NF, etc.) are essentially determined by dense layer pore size. As described in the literature, the pore size of dry-jet wet spun HFs can be influenced by air gap [16,17,18,19,20], water-soluble additives [21], non-solvent concentrations in the spinning solution [22], bore solution composition [16,23,24], coagulation bath composition [24], and thermal/chemical post treatment [23,25].

The air gap between the spinneret and coagulation bath is a key parameter contributing to pore size that can be easily varied during continuous fiber fabrication. In dry-jet wet spinning, the extruded nascent fibers undergo two physical changes in the air gap region: precipitation induced by moisture, and molecular reorientation caused by elongation stress [17]. Water vapor in the air is often a non-solvent component of spun polymer, and its slow phase separation may slightly reorient polymeric molecules and compress the free volume in outer dense layer when the air gap is increased. The elongation stress on nascent fiber is mainly a result of gravity on the fiber as vertically enters coagulation bath; thus, the elongation stress increases with the air gap. A moderate elongation stress is known to facilitate molecular orientation and reduce the dense layer porosity [17]. However, once the elongation stress is overcome, the phase-separated domains could be stretched apart to introduce more porosity. Because there is a threshold for the fiber orientation, the curved shape of the average pore size as a function of air gap could be completely different: when the spinning air gap is increased, membrane pore size may narrow and gas or water permeability may decline [17,18,26]. However, these effects are reported to vary, with some investigators obtaining opposite results [19] or noting that pore size may first decrease and then increase [20]. Therefore, the air gap effects must be determined on a case-by-case basis.

To our knowledge, the previous research regarding air gap effect is confined to a single specific application and mainly focused on fundamental aspects, such as membrane morphology [16,17,18,19,20], pore size distribution [19], surface roughness [16], fiber dimensional changes [15], and other study of probable contributors to such changes [17]. The focus of our work was to select and optimize a single practical parameter working window for the PBI HF fabrication, in which PBI HF membranes for RO, NF, or even ultrafiltration (UF) were prepared by merely varying the air gap. The resultant fiber products were characterized and evaluated in separation tests to indicate products with good potential for commercialization.

## 2. Experimental

### 2.1. Materials and Reagents

**Figure 1:**
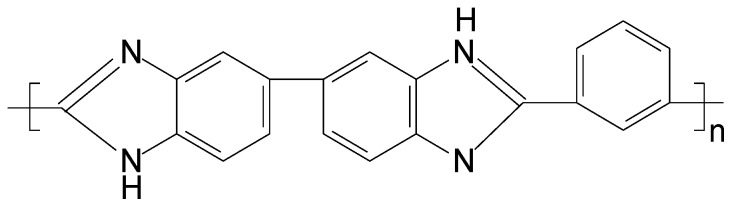
Chemical structure of poly[2,2′-(m-phenylen)-5,5′-bisbenzimidazole] (PBI).

The PBI adopted in this experiment is poly[2,2′–(m-phenylen)-5,5’-bisbenzimidazole] and its chemical structure is given in Figure 1. The PBI solution was supplied by PBI Performance Product and blended with dimethylacetamide (DMAc), polyvinylpyrrolidone (PVP), and isopropyl alcohol (IPA) to prepare dope solution for HF spinning. Bore solution is a mixture of non-solvents including methanol and IPA. A coagulation bath containing MeOH/IPA solution was used to form an outer dense layer of PBI HF through phase inversion. 10–15% sodium hypochlorite (NaClO) solution was diluted by deionized (DI) water to reduce the HF pore size, and the extra NaClO in HF was eliminated using sodium thiosulfate (Na_2_S_2_O_3_). Sodium chloride (NaCl), calcium chloride (CaCl_2_), magnesium sulfate (MgSO_4_), and sodium sulfate (Na_2_SO_4_), respectively, were dissolved in DI water to prepare feed solutions for membrane characterization. Sodium hydroxide (NaOH) solution and hydrochloric acid (HCl) solution were used to adjust pH value of feed solutions. The MgSO_4_ and CaCl_2_ were purchased from J.T. Baker Chemicals, the Na_2_SO_4_ and NaCl from Mallinckrodt, the IPA, and the MeOH were obtained from Macron Fine Chemicals, and the remaining items were supplied by Sigma-Aldrich (St. Louis, MO, USA).

### 2.2. Fabrication of PBI HF and Preparation of Fiber Module

**Figure 2:**
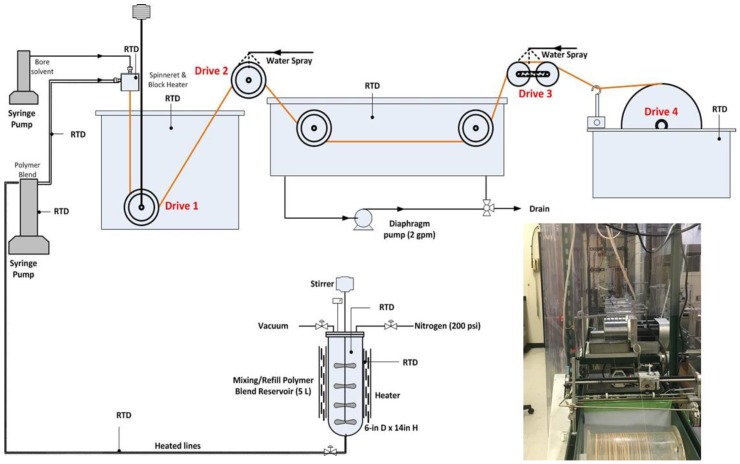
Schematic and photo image of spinning line. RTD is resistance thermometer.

The dry-jet wet spinning process for PBI HF fabrication is illustrated in Figure 2. A degassed PBI dope solution (15–20% in DMAc with viscosity in a range of 19,000–21,000 cP) was stored in a 5 L stainless reservoir and transferred to a syringe pump (1000D, Teledyne ISCO, Lincoln, NE, USA) before fabrication. The dope solution was extruded out of a spinneret at 35–55 °C and a flow rate of 1–2 cc/min. The HF lumen structure was kept by a non-solvent alcohol solution as the bore solution with a flow rate of 0.2–1 cc/min. The flow rates of both the dope solution and the bore solution were controlled by two syringe pumps, respectively. The air gap between the spinneret and coagulation bath was varied from 0.5 to 4 inches. A thin HF barrier layer was formed in the non-solvent alcohol coagulation bath at 5–15 °C followed by a washing in water bath at room temperature to remove chemical residuals and to conduct further phase inversion. The details of the spinning process can be found in the patent published by SRI International [27]. The HFMs collected on the take-up drum (Drive 4) were washed in a warm water bath to remove DMAc and other chemicals. The water bath was replaced by fresh water every 12 h to remove trace chemicals, and the waste water was monitored by an ultraviolet (UV)-visible spectrophotometer (8453, Hewlett Packard, Santa Clara, CA, USA) until no absorbance peak at 480 nm was detected.

The washed fibers were dried and crosslinked in the solution of 2–6% dibromobutane in methyl isobutyl ketone for 4–16 h at 100 °C. The crosslinked fibers were dried and thermal treated for 3–6 h at 150 °C to get rid of chemical residuals.

For the application of RO, PBI HF desalination performance can be further enhanced by a chemical post-treatment surface modification. We soaked PBI HFs fibers (air gap = 2.5″, 6.4 cm) were soaked in 1000 ppm NaClO solution for 1 h, washed them with 1 wt% Na_2_S_2_O_3_ to remove the residual NaClO, and flushed them in DI water.

The test modules were prepared by epoxy potting 100 or more fibers with a length of 40 cm that were randomly selected from a large pile of fibers. The fiber module construction was a dead-end design, and the effective surface area of each module was ~5.5 × 10^−2^ m^2^.

### 2.3. Scanning Electron Microscopy (SEM) Measurements

The PBI HFs were chilled and fractured in liquid nitrogen for cross-section observation. All the samples were coated by platinum sputtering and observed under field-emission scanning electron microscope (FE-SEM) (JEOL6700, JEOL Ltd., Peabody, MA, USA) in lower secondary electron (LEI) mode with an accelerating voltage of 3 KV and a probe current of 20 µA.

### 2.4. Desalination Test

**Figure 3:**
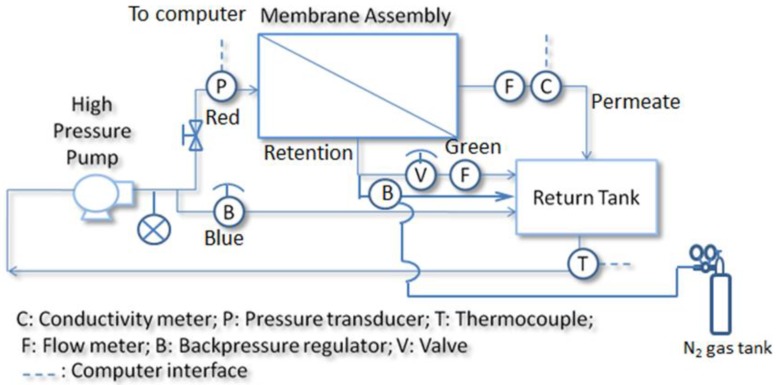
Process flow diagram (PFD) of the custom-built desalination system.

A custom-built filtration system was used to characterize desalination performance of 100-fiber PBI HF modules and its process flow diagram (PFD) is shown in Figure 3. A solution of 2000 ppm single salt (i.e., MgSO_4_ CaCl_2_, Na_2_SO_4_ or NaCl) was circulated in the system at a flow rate of 1 gallon per hour (GPH) and under a hydraulic pressure of 100–700 psi (6.9–48.3 bar). The solution temperature was stabilized at 25 ± 2 °C by a recirculating chiller (Thermo Neslab RTE-110, Thermo Fisher, Waltham, MA, USA). The pH value of the feed solution was adjusted using a 1 M NaOH or 1M HCl aqueous solution and monitored by a pH meter (PerpHecT LogR Orion 370, Thermo Scientific, Waltham, MA, USA). The PBI HF modules were equilibrated under a specific condition for 2 h before data collection. Permeate flux and conductivity were measured every 10 min using a 50-mL graduated cylinder and a conductivity meter (CON 110, Oakton Instruments, Vernon Hills, IL, USA), respectively. Salt rejection *R* (%) was calculated using the following equation:

(1) $$R=\left(1-\frac{C_{P}}{C_{F}}\right)\times 100\%$$

where *C_P_* and *C_F_* represent the solute concentrations of permeate solution and feed solution, respectively.

## 3. Results and Discussions

### 3.1. Morphology of PBI HFs

**Figure 4:**
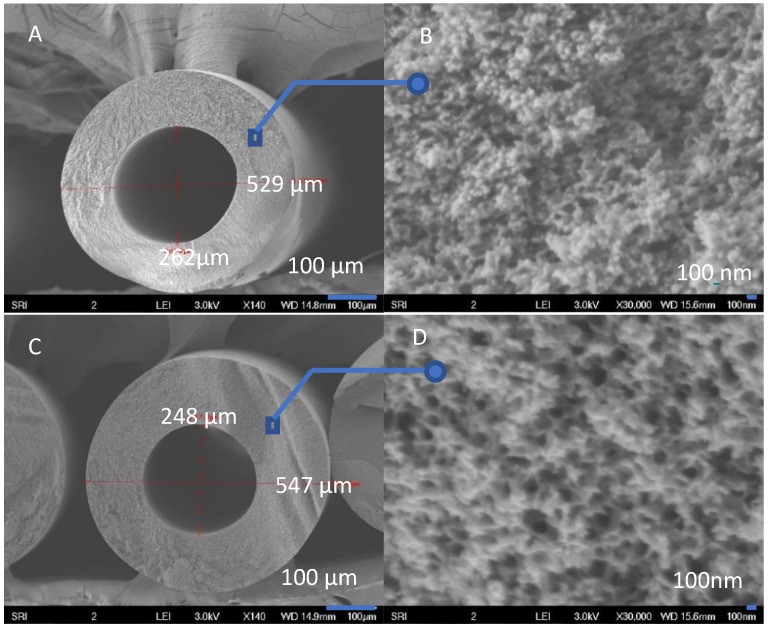
The cross-section morphology of PBI HFs with air gap of (**A**,**B**) 0.5″ (1.3 cm) and (**C**,**D**) 4″.

**Figure 5:**
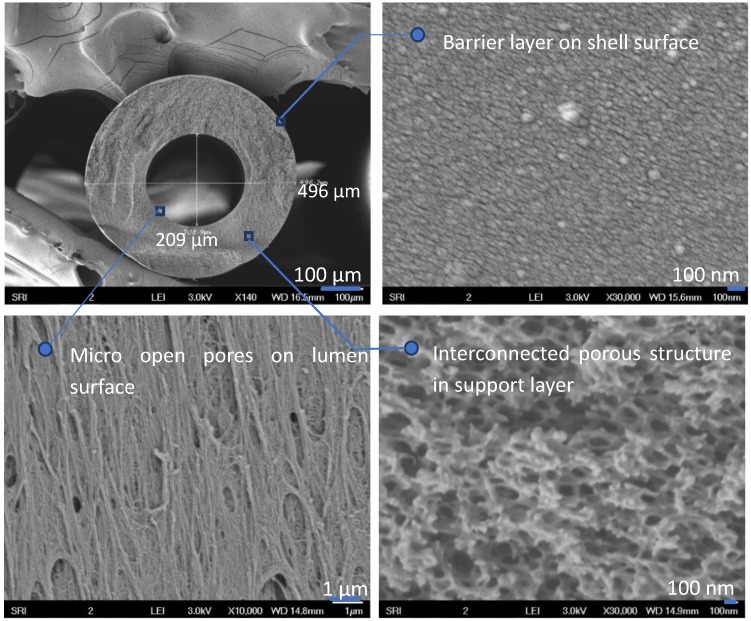
Typical morphology of polybenzimidazole hollow-fiber (PBI HF) (air gap = 2.5”).

As shown in Figure 4, the PBI HFs had similar cross-section morphology when the spinning air gap was varied from 0.5″ (1.3 cm) to 4″ (10.2 cm). As a demonstration, Figure 5 shows a typical morphology of PBI HFs (air gap = 2.5″, 6.4 cm) consisting of a dense barrier layer, a sponge-like substructure, and a porous inner layer. A nodular structure can be observed on outer dense layer, probably because of a metastable state with a non-uniform dispersion of polymer concentration during the liquid–liquid demixing in the coagulation bath. Some areas obtained concentrations above vitrification; these were quickly frozen by the non-solvent and led to a “frozen island” structure (i.e., nodule formation) [28]. The sponge-like substructure and porous lumen side formed due to the delayed phase separation [29]. The large open pores and the inter-connected pore structure can effectively increase the water flux with reduced pressure drops. There are no macrovoids in the cross section because the comparatively gentle coagulants (i.e., IPA and IPA/MeOH) were applied outside and inside the HFs [30]. A macrovoid-free structure guarantees adequate mechanical strength to sustain the high-pressure RO and NF tests.

**Figure 6:**
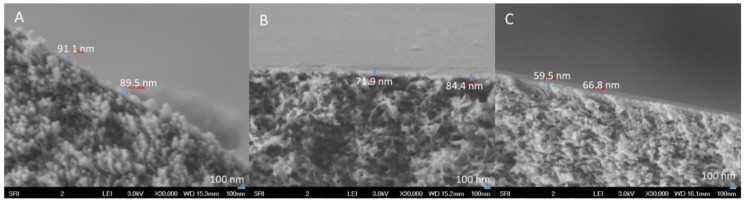
SEM images of PBI HF outer barrier layers spun with air gaps of (**A**) 0.5″ (1.3 cm), (**B**) 2.5″ (6.4 cm), and (**C**) 4″ (10.2 cm).

Figure 6 provides high-magnification SEM images of HF outer dense layers fabricated with different air gaps. All of them possess comparable dense-layer thicknesses of less than 100 nm. Because most of the pressure drop across the membrane is a function of the barrier layer, an asymmetric membrane structure with an ultra-thin barrier can effectively enhance the membrane separation efficiency.

**Figure 7:**
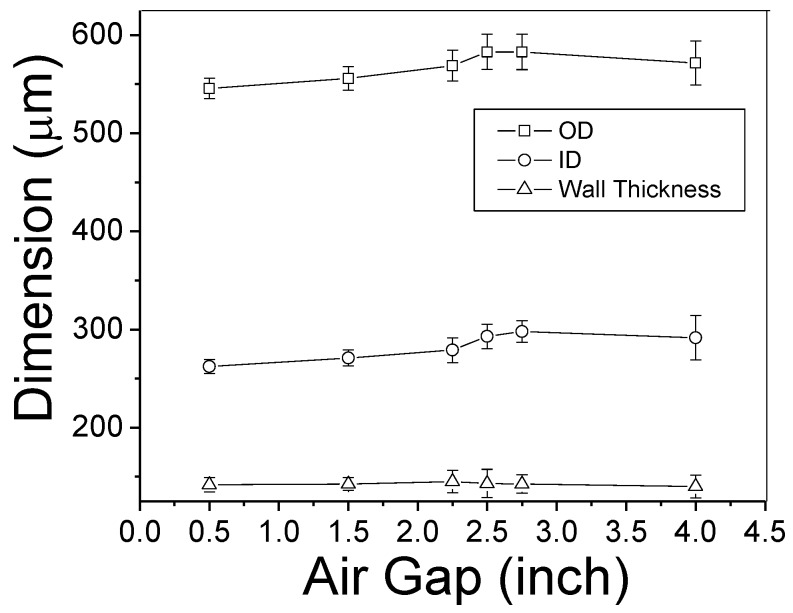
PBI HF dimensional change as a function of air gap.

Figure 7 depicts the HF dimensional change as the air gap increases. Each data point is based on 30° measurements of fiber cross section under a digital microscopy (VHX-600, Keyence, Itasca, IL, USA). There is no dramatic change caused by air gap in the fiber outer diameter (OD), inner diameter (ID), or wall thickness. Chung et al. built a mathematic model to fundamentally understand HF formation and to predict HF dimension as a function of air gap [18]. The ratio of OD and ID is mainly determined by the flow-rate ratio of the dope and bore solutions; both the OD and ID gradually declined as the air gap increased. In our experiment, only the air gap was adjusted and all the rest factors were kept constant for the fiber fabrication. Therefore, OD/ID changed negligibly when the air gap varied from 0.5″ (1.3 cm) (OD/ID = 2.08) to 4″ (10.2 cm) (OD/ID = 1.96). When the air gap was increased, both OD and ID increased rather than decreased by 26 µm and 29 µm, respectively, which disagrees with the previously reported model [18]. Nevertheless, the dimensional changes were comparable to the error bars (i.e., standard deviations) of OD and ID in Figure 7, and the air gap variation was much less than that cited by Chung et al. Thus, it is reasonable to conclude the dimensional changes caused by air gap is negligible as well.

### 3.2. RO Test

**Figure 8:**
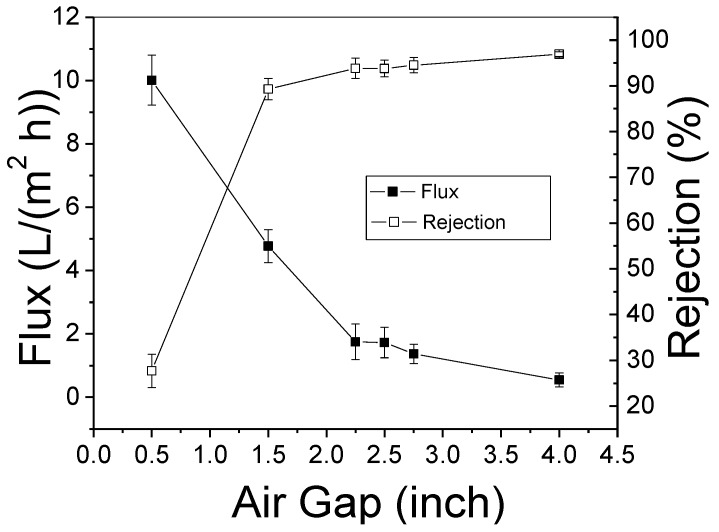
Desalination performance of PBI HF varied with air gap (test condition: 2000 ppm NaCl, 300 psi, and 25 ± 2 °C).

The desalination performance of PBI HF modules varied by air gap at 300 psi (20.7 bar) is shown in Figure 8. There were steep changes of both water flux and salt rejection in the range of 0.5–2.25″ (1.3–5.7 cm), which indicate gravity-induced elongation stress played the most important role by orienting and compacting polymer molecules to dispel free volume [15]. The SEM images in Figure 6 show that the membrane thicknesses were comparable when the air gap was varied from 0.5″ to 2.5″ (1.3–6.4 cm) and the Fick’s Law indicates that membrane flux is reversely proportional to its barrier layer thickness when the permeability and cross membrane pressure are fixed. But in Figure 8, the flux at 2.5″ (6.4 cm) is only about one fifth of that at 0.5″ (1.3 cm), which means the membrane permeability change contributes most of the declined flux. The permeability change was mainly caused by the further prove the declined water flux was mainly due to compaction of free volume (i.e., declined pore size) due to the increased salt rejection ratio rather than the change of dense layer thickness. The PBI HF with an air gap of 2.5″ (6.4 cm) has a water flux of 1.73 L·m^−2^ h^−1^ (LMH) and rejection of 93.8%. At an air gap of 4″ (10.2 cm), the rejection increased to just 96.9%, but the water flux decreased to 0.55 LMH. In the range of 2.25–4″ (5.7–10.2 cm), changes in rejection and flux were gradual, and no significant improvements in desalination performance were obtained by gently adjusting the air gap to 2.25″ (5.7 cm) and 2.75″ (7.0 cm). To balance the trade-off between flux and rejection, we selected the PBI HF with an air gap of 2.5" (6.4 cm) for the RO test.

**Figure 9:**
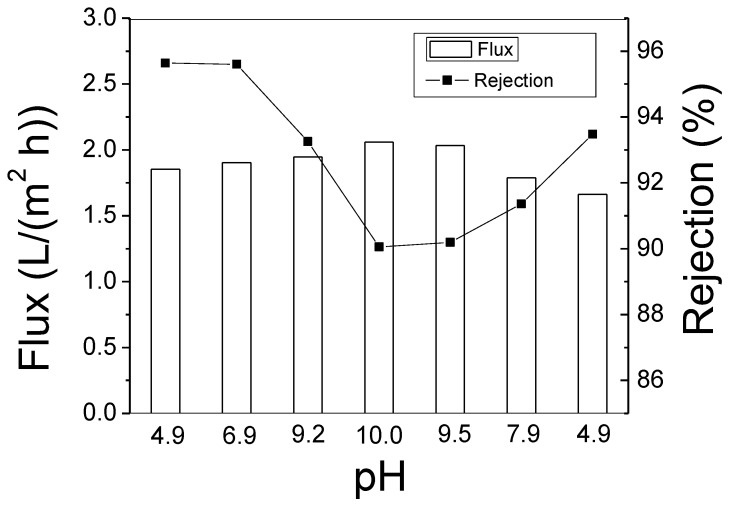
Rejection and flux of the PBI HF module (air gap = 2.5″, 6.4 cm) varied by solution pH (pH was adjusted by adding 1.0 mol/L NaOH or 1.0 mol/L HCl; the test conditions were 2000 ppm NaCl, 300 psi or 20.7 bar, and 25 ± 2 °C).

The desalination performance of PBI HF was sensitive to pH of the feed solution because the N-H functional groups in the imidazole ring have a lone pair on the nitrogen that acts as a proton acceptor. In an aqueous solution, the PBI membrane surface tends to be positively charged [5,31], and its rejection of salt is enhanced because it repels cations in the solution due to the Donnan exclusion effect. As is shown in Figure 9, the PBI HF rejects NaCl above 95% when the pH value is lower than 7. The surface-charge density is decreased when the solution becomes more basic because of the equilibrium for the ionization of the PBI N-H functional group in aqueous solution. Therefore, the salt rejection falls to 90% at pH 10. Because the surface-charge process is reversible, the PBI HF salt rejection improves when the pH is falls. The PBI HF has a good chemical resistance and works well in extremely acidic or basic aqueous solutions. The OH^−^ or H^+^ are smaller than salt ions, so the salt rejection deteriorates with overdoses of OH^-^ or H^+^ in the solution [32]. Therefore, the usefulness of varying the solution pH value is limited in the 4.9–10 range.

**Figure 10:**
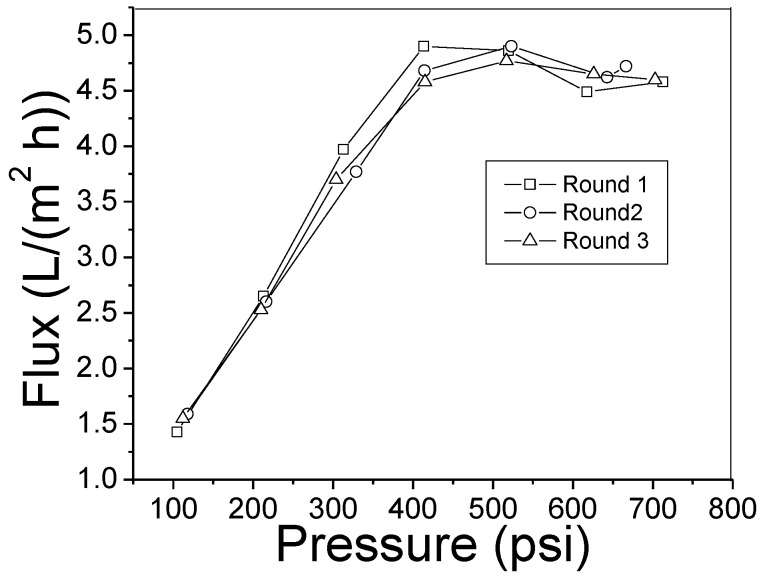
Pure water flux of PBI HF module (air gap = 2.5″, 6.4 cm) varied by hydraulic pressure.

The relationship of the PBI HF pure water flux against hydraulic pressure is given in Figure 10. There are linear and non-linear areas with a threshold at 400 psi (27.6 bar), which indicates the PBI HF is sensitive to high pressure. For the desalination of sea water, high hydraulic pressure is required to overcome osmosis pressure and drive water through membrane. In a typical RO membrane, water flux is well predicted by Fick’s law. As such, water flux is increased by pressure faster than salt flux; therefore, high salt rejection as well as high flux can be obtained [33]. The plateau curve above 400 psi (27.6 bar) for PBI HF implies the entire HF porous structure may be influenced by the high pressure as what occurred in other pressure-sensitive membranes [34]. Therefore, PBI HF RO membrane is only suitable for treating brackish water at a comparatively low pressure.

**Figure 11:**
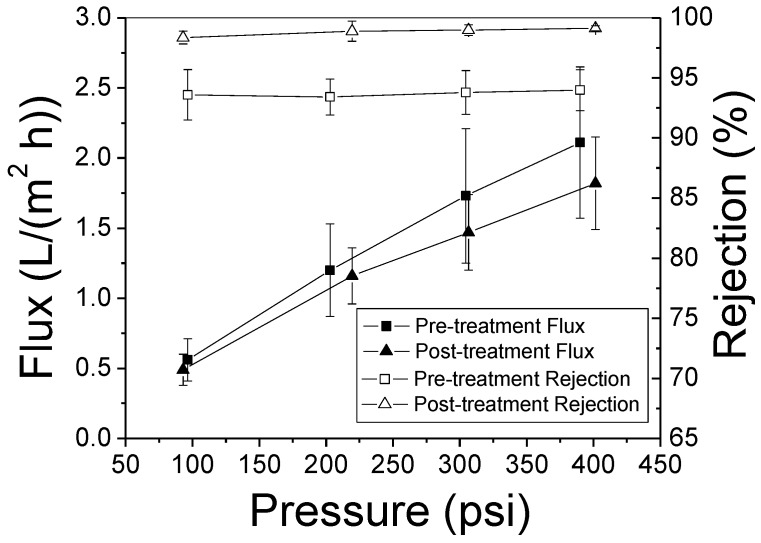
Desalination performance of PBI HF module (air gap = 2.5″, 6.4 cm) before and after surface modification (Test condition: 2000 ppm NaCl, pH 7.0 and 25 ± 2 °C).

The desalination performance before and after the surface modification is shown in Figure 11. At 300 psi (20.7 bar), the initial salt rejection and water flux rates were 93.8% and 1.73 LMH, respectively. After the post treatment, the salt rejection rate at 300 psi (20.7 bar) was boosted to 99.0% and the corresponding water flux decreased to 1.47 LMH. The PBI is known to possess a good anti-chlorine capability because of its molecular structure [31] and a technical report prepared by Celanese Research Company claims that PBI fibers retain their physical properties and do not suffer chemical degradation even when they are soaked in a 10-ppm free chlorine solution at pH 5.5 over 28 d [35]. However, the salt rejection achieved using NaClO solution in this experiment leads us to believe that PBI HF desalination performance may be influenced by chlorine. Few literatures with respect to the PBI membrane separation performance influenced by NaClO were published according to the authors’ knowledge, so the reason lead to the enhancement shown in Figure 11 is not clear currently. We intend to study on PBI chlorine resistance at later date.

**Figure 12:**
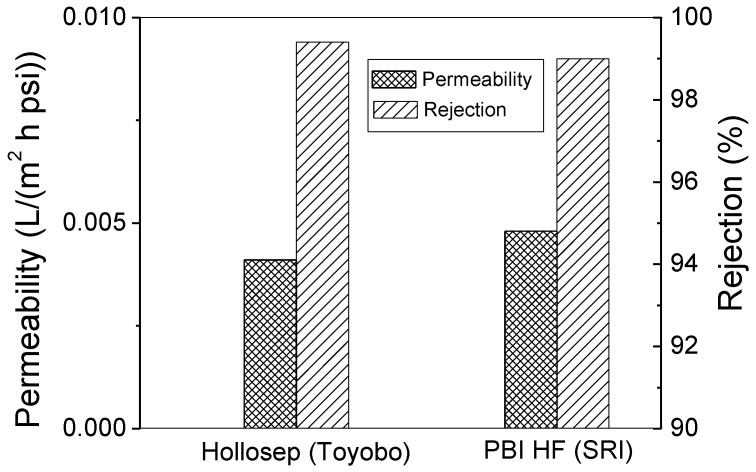
Comparison of desalination performance of modified PBI HF module (air gap = 2.5″, 6.4 cm) and a commercial HF product.

Compared to flat-sheet membranes, HFs usually have a lower water permeability (or A value). But this drawback can be compensated by the high packing density of HF module [10]. In terms of water flux per unit of module volume, an HF module should be comparable to a spiral-wound module. A comparison of the desalination performance of a modified PBI HF and a commercial HF module (made by Toyobo) is shown in Figure 12. The Toyobo product is Hollosep MH10255FI membrane, and its permeability is calculated according to the reported A value (1.5 × 10^−6^ cm^3^/cm^2^ s·[kg/cm^2^]) [36]. Both our modified PBI HF and the Hollosep provide over 99% salt rejection and their water permeability is comparable.

### 3.3. NF Test

**Figure 13:**
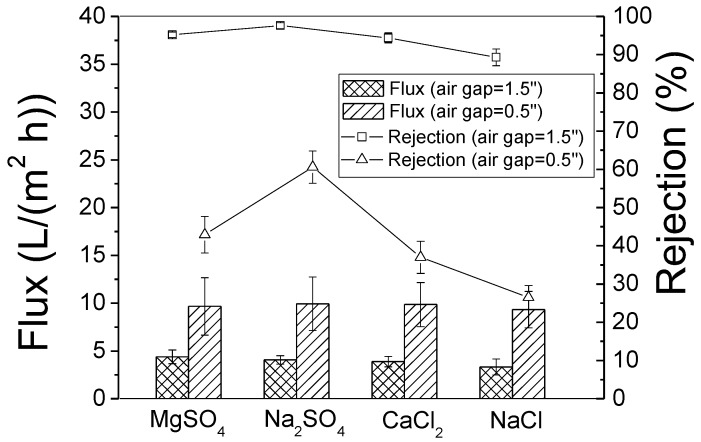
Salt rejection and water flux of PBI HF modules fabricated with different air gaps (1.5″ and 0.5″) or (3.8 cm and 1.3 cm) (Test condition: 2000 ppm single salt solution, 300 psi or 20.7 bar, pH 7.0 and 25 ± 2 °C).

When the air gap is decreased below 2.5″ (6.4 cm), the PBI HF barrier layer gains more free volume among molecular chains and, correspondingly, the membrane mean pore size should enlarge. This speculation is supported by the test result in Figure 8. Since PBI HF fabricated with air gap of 2.5″ (6.4 cm) has shown a comparable desalination performance to the commercial RO one, the next question is which air gap is suitable to fabricate NF membranes. As is shown in Figure 13, PBI HF fabricated with air gap of 1.5″ (3.8 cm) is thought to be a good NF candidate because it rejects both MgSO4 and Na_2_SO_4_ at >95%. For PBI HF with air gap of 0.5″ (1.3 cm), the pore size should be in the UF range because its rejection of salts containing multivalent ions is <60%. We will study PBI HF with an air gap = 0.5″ (1.3 cm) for UF uses future research.

The PBI HF with an air gap of 1.5″ (3.8 cm) rejects salts in the following sequence: MgSO_4_ ≈ Na_2_SO_4_ > CaCl_2_ > NaCl. The separation principle of the NF membrane can be explained by the sieving mechanism and Donnan effect [33]. According sieving mechanism principles, hydrated ions that are larger than the membrane pore size are rejected by the membrane barrier layer and the salt is rejected when either its cation(s) or anion(s) is rejected. Both Mg^2+^ and SO_4_^2−^ have the same hydrated radius (0.300 nm), while Ca^2+^ is smaller (0. 260 nm) [37]. The hydrated radii of Na^+^ and Cl^−^ are 0.178 nm and 0.195 nm, respectively, which are much smaller than other ions. The rejection sequence is well-explained by only the sieving mechanism. If the Donnan effect is strong enough, MgSO_4_ rejection should be significantly higher than that of Na_2_SO_4_ because Mg^2+^ will be strongly repelled by the positively charged surface. Although the existence of the positive surface charge in PBI HF is supported by the test results in Section 3.2, the NF test result indicates that the surface charge has a very limited effect on the selectivity of multivalent ions.

The PBI fabricated by Chung et al. for NF/FO filtration has permeability of 18 L/(m^2^·h·bar), which is higher than the PBI HF (air gap = 1.5″. 3.8 cm) in our work (5.5 × 10^−2^ L/(m^2^·h·psi) or 8 L/(m^2^·h·bar)). The permeability can be improved by increasing the flow rate of the bore solution during fiber spinning to moderately decrease the fiber wall thickness.

## 4. Conclusions

The asymmetric PBI HFMs with ultra-thin and defect-free outer dense layers were successfully fabricated in the continuous dry-jet wet spinning line developed by SRI International. The spinning air gap was adjusted from 4″ (10.2 cm) to 0.5″ (1.3 cm), and the fiber pore size was dramatically enlarged and correspondingly changed from poreless to even UF scale. Meanwhile, no significant changes in OD, ID, wall thickness and barrier layer thickness were observed, which is beneficial to the quality control for HF modules when the air gap is varied for a series of products. The PBI HF with an air gap of 2.5″ (6.4 cm) are sensitive to high pressure (>400 psi, 27.6 bar) and should be a good candidate for use in RO filtration of brackish fluid because the rejection rates are 93.8 ± 1.8% for 2000 ppm NaCl and the flux of 1.73 ± 0.48 LMH at 300 psi (20.7 bar). The post treatment conducted with HF membranes soaked in 1000 ppm NaClO solution for 1 h yielded good separation performance with pure water permeability of 4.8 × 10^−3^ LMH/psi (0.07 L/(m^2^·h·bar) and salt rejection of 99.0%—these results are comparable to those of a commercial HF product provided by Toyobo. The PBI HF RO performance was sensitive to solution pH value, and a lower pH (<7) led to a higher salt rejection because the membrane’s surface charge density was increased by the ionization of N-H in the PBI imidazole ring. The PBI HFMs with an air gap of 1.5″ (3.8 cm) are suitable for NF and have high rejection rates for MgSO_4_ (95.2 ± 0.72%) and Na_2_SO_4_ (97.6 ± 0.43%). Testing in different feed solutions showed the positive surface charge in PBI HFs seemed to have little effect on the rejection of multivalent ions. The water permeability of PBI HF NF was 8L/(m^2^·h·bar), which is less than the reference data, leaving room for improvement.
